# Supplementary material for: The unsolved problem of otitis media in indigenous populations: a systematic review of upper respiratory and middle ear microbiology in indigenous children with otitis media
Source: Microbiome. 2018 Nov 5;6:199. doi: 10.1186/s40168-018-0577-2 (PMC6219068; doi:10.1186/s40168-018-0577-2)
Supplement: Supplementary file 1 — Search strategy. (DOCX 13 kb) [file 40168_2018_577_MOESM1_ESM.docx]

**Additional File 1: Search Strategy**

S1. MH microbiota OR MW “microbiology” OR microbio* OR bacteria* OR vir* OR protozoa OR fung*

S2. Upper AND ((respiratory OR airway) N2 tract) OR Nose OR mouth OR oral OR oropharynx OR tonsil OR adenoid* OR nasopharynx OR gum* OR nasal OR Immunity OR pathogenesis OR epidemiolog*

 S3. “Glue ear” OR “otitis media” OR MH "Otitis Media+" OR ear N5 (infection* OR disease) OR ear

S4. Indigenous OR aborigin* OR Maori OR Inuit OR “Native American“OR “Native americans” OR "first nation” OR "first nations" OR tribe OR tribes OR (ABENAKI OR pima* OR navajo* OR inuit* OR cherokee* OR shawnee* OR lakota OR ((ute OR utes OR biloxi OR blackfoot* OR apache* OR cheyenne OR spokane OR TUSCARORA OR tunica OR dakota OR sac OR fox OR creek* OR deleware* OR iowa* OR miami* OR mission* OR sioux OR omaha) AND (Indian OR Indians OR aborigin* OR "first nation" OR "first nations" OR indigenous OR tribe OR tribes OR tribal)) OR Coushatta OR tlingit* OR arapaho* OR Assiniboine OR BEOTHUK OR blackfeet OR blackfoot OR cabazon* OR CADDO* OR CHICKASAW OR CHIPPEWA OR CHITIMACHA* OR CHOCTAW* OR COCOPAH* OR "COEUR D'ALENE" OR COMANCHE* OR Muscogee* OR duwamish OR elwha* OR flathead* OR GOSHUTE* OR HO-CHUNK* OR hopi OR hopis OR hoopa* OR S'KLALLAM OR JATIBONICU OR JUMANO* OR kalapuya* OR kiowa* OR KOOTENAI* OR lemhi* OR shoshone* OR makah* OR pequot* OR MECHOOPDA* OR metis OR menominee* OR MICCOSUKEE* OR mi'kmaq OR mohegan* OR MUSCOGEE* OR navajo* OR "nez perce" OR "nez pierce" OR oneida* OR osage* OR passamaquoddy OR pawnee* OR "pend d'oreille" OR pomo OR pomos OR POTAWATOMI* OR pueblo* OR QUINAULT* OR salish OR saponi OR saponis OR SEMINOLE* OR shawnee* OR shoshone* OR siletz OR nakota OR S'KLALLAM OR suquamish OR taino* OR tohono OR o'odham OR tunica-biloxi OR tunicas OR umatilla* OR umpqua* OR waccamaw* OR wampanoag* OR washoe* OR wiyot* OR yakama*a

S5. S1 AND S2 AND S3 (all titles checked)

S6. S5 AND S4

Limit: all child
