# Supplementary material for: The unsolved problem of otitis media in indigenous populations: a systematic review of upper respiratory and middle ear microbiology in indigenous children with otitis media
Source: Microbiome. 2018 Nov 5;6:199. doi: 10.1186/s40168-018-0577-2 (PMC6219068; doi:10.1186/s40168-018-0577-2)
Supplement: Supplementary file 2 — Summary of OM diagnostic criteria used in studies. (DOCX 12 kb) [file 40168_2018_577_MOESM2_ESM.docx]

**Additional File 2: Summary of OM diagnostic criteria used in studies**

| Type of OM | Definition |
| --- | --- |
| Acute otitis media (AOM) without perforation | Moderate or marked bulging of TM/ any bulging without perforation  AND type B tympanogram  OR decreased mobility of TM on pneumatic otoscopy  OR serous or purulent ear discharge in external auditory meatus or red, grey, yellowish bulging of TM and absence of normal landmarks in patients with concurrent symptoms (e.g. otalgia, fever, irritability) |
| AOM with perforation (AOMwP) | Middle ear discharge for < 6 weeks and TM perforation covering <2% of pars tensa  OR Middle ear discharge observed and TM perforation recently healed |
| Otitis media with effusion (OME) | TM from normal🡪 mildly bulging  OR middle ear effusion behind an intact TM identified by an air-fluid level/ bubble  AND a type B tympanogram  OR decreased mobility of TM on pneumatic otoscopy  ± absent stapedial reflex  ± abnormal audiometry of ≥25DB on at least 2 frequencies  ± air-bone gap on audiometry with conductive hearing loss of >20BD  ± without signs of acute infection or recent perforation |
| Chronic suppurative otitis media (CSOM) | Middle ear discharge for >6 weeks and TM perforation covering >2% of the pars tensa  OR middle ear discharge >2 weeks and a TM perforation  OR TM perforation covering >2% of pars tensa and middle ear discharge |

TM, tympanic membrane
