# Supplementary material for: The unsolved problem of otitis media in indigenous populations: a systematic review of upper respiratory and middle ear microbiology in indigenous children with otitis media
Source: Microbiome. 2018 Nov 5;6:199. doi: 10.1186/s40168-018-0577-2 (PMC6219068; doi:10.1186/s40168-018-0577-2)
Supplement: Supplementary file 3 — Summary of three main otopathogens in indigenous children with otitis media. (DOCX 32 kb) [file 40168_2018_577_MOESM3_ESM.docx]

**Additional File 3:** **Summary of three main otopathogens in indigenous children with otitis media**

| Study | Reference | | Type of OM | Sample site | | *H. influenzae*  n (%) | *S. pneumoniae*  n (%) | | *M. catarrhalis*  n (%) | | >2 otopathogens  n (%) | | 2 otopathogens  n (%) | Culture vs PCR | |  |
| --- | --- | --- | --- | --- | --- | --- | --- | --- | --- | --- | --- | --- | --- | --- | --- | --- |
| Acute Otitis Media - Australian Indigenous | | | | | | | | | | | | | | | | |
| 2011, Binks^ | [35] | | AOM | NP | | 110 (96) | 109 (95) | 113 (98) | | | - | | - | | PCR | |
|  |  | | AOMwP | NP | | 52 (90) | 52 (90) | 57 (98) | | | - | | - | |  | |
| 2005, Gibney^ | [50] | | AOM/  AOMwP | NP | | 66 (71) | 76 (82) | 86 (96) | | | 59 (63) | | - | | Culture | |
|  |  | |  | MED | | 12 (32) | 11 (29) | 2 (38) | | | 1 (3) | | - | |  | |
| 2006, Leach^ | [51] | | AOMwP | MED | | 78 (57) | 46 (34) | 5 (4) | | | - | |  | | Culture | |
| 2009, Mackenzie^ | [52] | | AOMwP | MED | | 49 (48) | 35 (34) | 2 (2) | | | - | | 27 (26) | | Culture | |
| 2010, Morris | [53] | | AOMwP | Nose | | 269 (85) | 267 (84) | - | | | - | | - | | Culture | |
|  |  | |  | MED | | 27 (39) | 19 (27) | - | | | - | | - | | Culture | |
| 2013, Smith-Vaughan | [48] | | AOMwP | NP | | 45 (82) | 46 (84) | 51 (93) | | | 40 (73) | | - | | Culture  PCR | |
|  |  | |  | MED | | 49 (89) | 23 (41) | 10 (18) | | | 3 (5) | | - | | Culture  PCR | |
| Acute Otitis Media - Greenlandic Inuit | | | | | | | | | | | | | | | | |
| 1996, Homøe | [34] | | AOM/  AOMwP | NP | | 44 (81) | 26 (48) | | 27 (50) | | 42 (78) | | - | Culture  PCR | |  |
|  |  | |  | MED | | 12 (50) | 13 (54) | | 4 (17) | | 11 (46) | | - | Culture PCR | |  |
| Otitis Media with Effusion – Australian Indigenous | | | | | | | | | | | | | | | | |
| 2007, Ashhurst-Smith | | [37] | OME | | MEE | 0 | 0 | | | 0 | | NR |  | Culture PCR | |  |
| 2011, Binks^ | | [35] | OME | | NP | 155 (89) | 155 (89) | | | 167 (95) | | NR | - | PCR | |  |
| 2015, Jervis-Bardy | | [57] | OME | | NP | 10 (91) | -* | | | 11 (100) | | 11 (100) |  | 16S rRNA PCR | |  |
|  | |  |  | | MEE | 5 (63) | -* | | | 2 (25) | | 8 (100) | - |  | |  |
| 2008, Leach^ | | [54] | OME | | NP | 79 (77) | 82 (80) | | | 80 (78) | | 55 (53) | - | Culture | |  |
| 2003, Stuart | | [49] | OME | | MEE | 3 (7) | 0 | | | 2 (4) | | NR | - | Culture | |  |
| Otitis Media with Effusion – Greenlandic Inuit | | | | | | | | | | | | | | | | |
| 2009, Homøe | [39] | | OME | MEE | | 0 | 0 | | 0 | | 0 | | - | Culture | |  |
| Otitis Media with Effusion – Alaskan Inuit | | | | | | | | | | | | | | | | |
| 1999, Parkinson | [38] | | OME | MEE | | 44 (21) | 17 (8) | | 6 (4) | | - | | - | Culture | |  |
| Chronic Suppurative Otitis Media – Australian Indigenous | | | | | | | | | | | | | | | | |
| 2008, Leach | [43] | | CSOM | MED | | 21 (23) | 3 (3) | | | - | | - | - | Culture | |  |
| 2013, Stephen | [42] | | CSOM | NP | | 45 (52) | 61 (70) | | | 34 (39) | | - | - | Culture | |  |
|  |  | |  | MED | | 22 (43) | 5 (9) | | | 0 | | - | - | Culture | |  |
| Chronic Suppurative Otitis Media – Greenlandic Inuit | | | | | | | | | | | | | | | |  |
| 2009, Homøe | [39] | | CSOM | MED | | 0 | 0 | | 0 | | - | | - | Culture | |  |
| Unspecified Otorrhoea – Australian Indigenous | | | | | | | | | | | | | | | |  |
| 2015, Leach | [47] | | CSOM/ AOMwP | MED | | 58 (45) | 28 (20) | | 5 (4) | | - | | 17(14) | Culture | |  |
| 2016, Leach | [17] | | CSOM/ AOMwP | MED | | 30 (41) | 16(22) | | 6 (8) | | - | | 13(18) | Culture | |  |

Note: AOM, acute otitis media; AOMwP, acute otitis media with perforated tympanic membrane; CSOM, chronic suppurative otitis media; MED, middle ear discharge; MEE, middle ear effusion; NP, nasopharynx; OME, otitis media with effusion.

^ Overlapping participants. * *S. pneumoniae* were not specifically sought, however Mitis Group Streptococcus (inclusive of *S. pneumoniae* were reported in 10 (91%) of participants.
