# Supplementary material for: The unsolved problem of otitis media in indigenous populations: a systematic review of upper respiratory and middle ear microbiology in indigenous children with otitis media
Source: Microbiome. 2018 Nov 5;6:199. doi: 10.1186/s40168-018-0577-2 (PMC6219068; doi:10.1186/s40168-018-0577-2)
Supplement: Supplementary file 4 — Summary of microorganisms reported by studies of URT and/or middle ear specimens using specialist laboratory methods. (DOCX 35 kb) [file 40168_2018_577_MOESM4_ESM.docx]

**Additional File 4: Summary of microorganisms reported by studies of URT and/or middle ear specimens using specialist laboratory methods**

| Study | Reference | Type of OM | Sample type | Bacteria, n(%) | | | | | | | Viruses, n(%) | | | | | | | Yeast/ Fungi, n(%) |
| --- | --- | --- | --- | --- | --- | --- | --- | --- | --- | --- | --- | --- | --- | --- | --- | --- | --- | --- |
|  |  |  |  | *Staphylococcus* spp. | Β haemolytic streptococcus | *A. otitidis* | *Pseudomonas* spp. | *Corynebacterium* spp. | *Chlamydia* spp. | Bocavirus | | Polyomavirus | Adenovirus | Rhinovirus | Coronavirus | Enterovirus | Influenza B |  |
| Australian Indigenous | | | | | | | | | | | | | | | | | | |
| 2011, Binks | [35] | AOM | NP | - | - | - | - | - | ^-^ | 10(9) | | 19(17) | 22(19) | 47(41) | 5(4) | - | - | - |
| 2011, Binks | [35] | AOMwP | NP | - | - | - | - | - | ^-^ | 4(7) | | 5(9) | 13(22) | 18(31) | 2(3) |  | - | - |
| 2012, Marsh | [56] | AOMwP | NP | - | - | 0(0) | - | - | - | - | | - | - | - | - | - | - | - |
| 2012, Marsh | [56] | AOMwP | MED | - | - | 10(37) | - | - | - | - | | - | - | - | - | - | - | - |
| 2006, Leach | [51] | AOMwP | MED | 84(62) | 8(6) | - | - | - | - | - | | - | - | - | - | - | - | - |
| 2013, Smith-Vaughan | [48] | AOMwP | MED | - | 5(9) | - | - | - | - | - | | - | - | - | - | - | - | - |
| 1985, Dawson | [55] | OME | MEE | 1(3) | - | - | 1(3) | - | 2(7) | - | | - | - | - | - | - | - | - |
| 2003, Stuart | [49] | OME | MEE | 5(11) | - | - | 1(2) | 4(9) | - | - | | - | - | - | - | - | - | 1(2) |
| 2007, Ashhurst-Smith | [37] | OME | MEE | - | - | 10(45) | 1(5) | 8(36) | ^-^ | - | | - | - | - | - | - | - | - |
| 2011, Binks | [35] | OME | NP | - | - | - | - | - | ^-^ | 14(8) | | 20(11) | 13(7) | 69(39) | 7(4) | - | - | - |
| 2003, Couzos | [40] | CSOM | MED | 20(15) | - | - | 59(44) | - | - | - | | - | - | - | - | - | - | 5(4) |
| 2008, Leach | [43] | CSOM | MED | - | - | - | 58(62) | - | - | - | | - | - | - | - | - | - | 18(19) |
| 2013, Stephen | [42] | CSOM | NP | 13(15) | - | - | - | - | - | - | | - | - | - | - | - | - | - |
| 2013, Stephen | [42] | CSOM | MED | 13(15) | 4(7) | - | 13(24) | - | - | - | | - | - | - | - | - | - | - |
| 1975, Copeman | [44] | CSOM/AOMwP | MED | 2(8) | 1(3) | - | - | - | - | - | | - | - | - | - | - | - | - |
| 1972, Stuart | [46] | CSOM/AOMwP | MED | 2(20) | - | - | 2(20) | - | - | - | | - | - | - | - | - | - | - |
| 1975, Stuart | [45] | CSOM/AOMwP | MED | 10(19) | 7(13) | - | 7(13) | - | - | - | | - | - | - | - | - | - | - |
| 2015, Leach | [57] | CSOM/AOMwP | MED | 68(49) | - | - | - | - | - | - | | - | - | - | - | - | - | - |
| 2016, Leach | [17] | CSOM/AOMwP | MED | 25(34) | - | - | - | - | - | - | | - | - | - | - | - | - | - |
| Greenlandic Inuit | | | | | | | | | | | | | | | | | | |
| 1996, Homøe | [34] | AOM  AOMwP | NP | 5(9) | 6(11) | - | - | - | 3(6) | - | | - | - | 11(28) | - | 12(31) | 1(2) | - |
| 1996, Homøe | [34] | AOM  AOMwP | MED | 4(17) | 4(17) | - | - | - | 0(0) | - | | - | - | 1(7) | - | 3(21) | 1(5) | - |
| 2009, Homøe | [39] | OME | MEE | 2(29) | - | - | - | - | - | - | | - | - | - | - | - | - | - |
| 2009, Homøe | [39] | CSOM | MED | 4(66) | - | - | - | - | - | - | | - | - | - | - | - | - | - |
| Alaskan Inuit | | | | | | | | | | | | | | | | | | |
| 1999, Parkinson | [38] | OME | MEE | 1(0.5) | 1(0.5) | - | - | - | - | - | | - | - | - | - | - | - | - |

n = the number of positive specimens

AOM, acute otitis media; AOMwP, acute otitis media with perforated tympanic membrane; CSOM, chronic suppurative otitis media; MED, middle ear discharge; MEE, middle ear effusion; NP, nasopharynx; OME, otitis media with effusion. – indicates that testing for this taxa was not performed.
