# Supplementary material for: The unsolved problem of otitis media in indigenous populations: a systematic review of upper respiratory and middle ear microbiology in indigenous children with otitis media
Source: Microbiome. 2018 Nov 5;6:199. doi: 10.1186/s40168-018-0577-2 (PMC6219068; doi:10.1186/s40168-018-0577-2)
Supplement: Supplementary file 5 — Summary of microorganisms identified in the nasopharynx/middle ear using next generation sequencing. (DOCX 14 kb) [file 40168_2018_577_MOESM5_ESM.docx]

## Additional File 5: Summary of microorganisms identified in the nasopharynx/ middle ear using next generation sequencing

| Study | Reference | Type of OM | Site | Other microorgansims  n (%) | Analysis technique |
| --- | --- | --- | --- | --- | --- |
| Australian Indigenous | | | | | |
| 2015, Jervis-Bardy | [57] | OME | Nasopharynx | *Bergeyella* 2 (18)  *Corynebacterium* 7 (64)  *Dolosigranulum* 11 (100)  Other *Haemophilus* 4 (36)  *Fusobacterium* 2 (18)  Other *Moraxella* 11 (100)  *Orthinobacterium* 6 (55)  *Porphyromonas* 4 (36)  *Prevotella* 4 (36)  *Streptococcus* 10 (91)  *Suttonella* 1 (9) | 16S rRNA gene sequencing |
|  |  |  | Middle ear effusion | *Alloiococcus* 5 (63%)  *Corynebacterium* 4 (50%)  *Enterobacter* 3 (38%)  *Staphylococcus* 2 (25%)  *Streptococcus* 3 (38%)  *Turicibacter* 1 (12.5%)  *Turicella* 4 (50%) |  |

OME, otitis media with effusion.
